# Portuguese Caregivers of Persons With Alzheimer’s Disease in the Context of the COVID-19 Pandemic: A Qualitative Study of the Grieving Process

**DOI:** 10.1177/00302228241246417

**Published:** 2024-05-02

**Authors:** Laura Brito, Ana Cristina Bernardo, Ângela Leite, M. Graça Pereira

**Affiliations:** 1 Psychology Research Centre, School of Psychology, 56059 University of Minho, Braga, Portugal; 2 Centre for Philosophical and Humanistic Studies, Portuguese Catholic University, Braga, Portugal

**Keywords:** alzheimer disease, caregiving, COVID-19, grief, end-of-Life

## Abstract

This study addresses the experiences of informal caregivers caring for elderly family members with Alzheimer’s disease (AD) during the COVID-19 pandemic. The study includes a sample of eight informal caregivers who were evaluated through a semi-structured interview six months after the death of a loved one they cared for. A thematic content analysis was applied to the interviews and yielded two higher-order domains: (1) Experiencing the AD and the death of a family member and (2) The grieving process during the COVID-19 pandemic. The results provide valuable insights into family caregivers' experiences regarding the profound emotional impact of caregiving and grief during the COVID-19 pandemic. Caregivers maintain their identity even after loss, underscoring the enduring impact of caregiving. The identity as a caregiver impacted coping and grief responses, highlighting the need for tailored interventions.

## Introduction

The aging population in Portugal is growing (National Statistical Institute, 2023), and the increasing prevalence of dementia places significant challenges to society. This condition results in limitations in daily functioning, requiring assistance from caregivers, particularly for the 10% of individuals aged over 65 and the 50% aged over 90 who are diagnosed with dementia (Alzheimer’s Association, 2021). In Portugal, the estimated number of informal caregivers (IC(s)) has reached 1.4 million, driven by the pandemic-induced closure of social services (Caring for Informal Caregivers Movement, 2021). However, it remains uncertain how many IC(s) specifically care for a person with Alzheimer's Disease (AD). Approximately 82% of Portuguese caregivers provide daily care to family members, being primarily women (64%) aged between 15 and 54 (Organization for Economic Cooperation & Development, 2019). The caregiving role is often influenced by several factors, including a sense of obligation shaped by societal norms, a desire to contribute to the well-being of the person with AD, emotional bonds, a sense of duty, compassion, coerced altruism, or altruistic motives (Costa et al., 2019; Caring for Informal Caregivers Movement, 2021). In Portugal, there is a strong tradition of keeping older family members at home, providing care and support within the family structure. Despite the emotional and physical challenges associated with AD caregiving, family members willingly embrace the role of caregivers, driven by a sense of duty, familial responsibility, and respect for the elderly (Oliveira et al., 2024).

The global estimate of dementia cases is as high as 24 million, and projections indicate that this number will double every 20 years until 2040 (Sleeman et al., 2019). During the early stages of the Coronavirus disease-2019, in Portugal, the first positive cases were reported on March 2, 2020, followed by the first death on March 16 (Chaíça, 2020). The State of Emergency required the suspension of citizens' rights to implement several public health protection measures, such as mandatory home confinement, sanitary cordons, and the closure of establishments (Peixoto et al., 2021). By the end of March 2020, the country recorded 7443 confirmed cases and 160 deaths (Epidemiologic Report in Portugal, 2020a). The period from October 24 to December 18, 2020 (Epidemiologic Report in Portugal, 2020b) marked the “second wave” in Portugal, reaching a peak of 7497 new cases on November 4, 2020 (Peixoto et al., 2021). Between December 19, 2020, and February 20, 2021, Portugal faced its highest systemic risk perception during the first year of the pandemic (Epidemiologic Report in Portugal, 2021). This period was characterized by the “third wave” culminating in 16,432 new cases on January 28, 2021. Media reports described Portugal as one of the worst-hit countries globally, with weekly averages of 13,200 cases and over 200 deaths per day, reaching peaks of over 300 deaths per day (Peralta-Santos et al., 2021).

The COVID-19 pandemic has also resulted in a higher mortality rate among older individuals. The new circumstances of the dying rituals and the restrictions implemented to contain the spread of the virus have further affected the grief and bereavement experiences of IC(s) who were unable to visit or were isolated due to these restrictions (Skantharajah et al., 2021). The lack of social support during mourning and the absence of traditional parting ceremonies negatively affected caregivers' lives, leading to conditions such as prolonged grief disorder, and post-traumatic stress disorder with a great impact on mental health (Laranjeira et al., 2022; Skantharajah et al., 2022). The Portuguese population reported difficulties in managing mourning and dissatisfaction with the inability to pay tribute to loved ones. Social support was found to be beneficial in coping with grief; however, Cunha et al. (2023) identified a positive association between avoidance strategies and prolonged grief disorder, suggesting the use of these coping mechanisms in the development of pathologic grief.

Grief and bereavement are common experiences for caregivers of individuals with AD and other forms of dementia (Manevich et al., 2023). IC(s), such as family members, often experience prolonged grief and stress due to the progressive nature of the disease and the significant changes in the person they are caring for (Dehpour & Koffman, 2023), with a detrimental impact on their physical and mental well-being (Crawley et al., 2022).

AD is marked by a cognitive and functional decline, which disrupts individuals’ daily activities and is frequently accompanied by changes in personality and behavior to the extent that most diagnosed individuals need or will eventually require some form of external care (Manevich et al., 2023; Rubin et al., 2019). Previous research indicates that caregivers of individuals with AD often confront a myriad of challenges, encompassing behavioral and psychological issues that are a consequence of the dementia process (Yuan et al., 2021), financial constraints (Liu et al., 2019), substantial workloads related to assisting with daily activities (Zenker et al., 2017), and isolation stemming from extended caregiving hours (Victor et al., 2021) that may lead to adverse outcomes such as caregiving burden (Yuan et al., 2020). The emotional toll of caring for someone with AD is associated with an elevated risk of depression, anxiety, and other stress-related disorders. The complexities of caregiving, compounded by the eventual loss and death of the person living with AD, contributed to these adverse effects (Breen et al., 2018; Washington et al., 2018). Nevertheless, beyond the challenges, IC(s) also encounter positive aspects while tending to their loved ones, and these positive dimensions significantly influence the caregiver’s stress and overall well-being (Yu et al., 2018). In a recent review focusing on the favorable aspects of caregiving, four primary domains emerged such as a sense of personal accomplishment and fulfillment, feelings of mutual support in the dyadic relationship, higher family cohesion and functionality, and a perception of personal growth and purpose in life (Yu et al., 2018).

Severe AD brings about substantial suffering, affecting those afflicted by the condition and their family caregivers, especially as the illness progresses. Evaluating the suffering becomes notably intricate in such cases, given the limited capacity of individuals with severe AD to express their distress (Malhotra et al., 2021). However, understanding and measuring suffering within the context of grief remains essential for enabling person-centered care for individuals with severe AD (Malhotra et al., 2021).

The beginning of the end-of-life phase remains ambiguous, in the dementia process. However, families are universally tasked with increasingly intricate decisions as the disease advances (Browne et al., 2021; Jones et al., 2019) leading family caregivers through multifaceted transitions spanning occupational, environmental, and relational roles, with an impact on their physical, emotional, and mental well-being (Kokorelias et al., 2021). The end-of-life phase is a transitional period, where caregiver preparedness for death and social support help in the grief process are essential (Moore et al., 2017, 2020). Nishimura et al.’s study (2022) underscored the importance of the relationship between caregiver-person with AD in facilitating a positive end of life, with Portugal prioritizing home-based care. Given the family’s central role in dementia care and the need for guidance and support amidst disease progression (Colussi et al., 2019), understanding the end-of-life significance in family caregivers, within a pandemic context, becomes essential.

The terms grief, bereavement, and mourning are often used interchangeably when describing the experiences of informal caregivers. However, the terms must be clarified: while grief is seen as a process, bereavement refers to a specific period during which grief is experienced, and mourning involves the outward expression of grief through social and cultural practices (Thrower et al., 2023). Grief is a multifaceted process characterized by a range of symptoms and manifestations encompassing emotional changes such as sadness, alterations in appetite, and heightened vulnerability to psychiatric conditions such as major depressive disorder (Rupp et al., 2023). Moreover, the grieving journey entails a complex interplay of emotions, including ambiguity, disenfranchised grief, a sense of loss of companionship, autonomy, and authority, anger and guilt. However, grief also involves psychosocial adaptation and coping mechanisms (Blandin & Pepin, 2017).

Different types of grief, such as anticipatory grief occurs before a loss and involves anticipating what will be lost; while complicated grief is prolonged and impairs the individual’s functioning (Cheung et al., 2018; Coelho et al., 2018; Dehpour & Koffman, 2022). In addition, caregivers may experience anticipatory grief as they witness the decline of their loved one’s cognitive and functional abilities. The grief of a caregiver usually consists of a pre-morbid state, followed by the onset of grief that precipitates multiple and ambiguous losses that, in turn, may lead to difficulties in adapting to the grieving process. For most caregivers, the most significant loss occurs after the death of the loved one since it implies the loss of the caregiver role. The suffering is so intense that the death of the person living with AD may also bring feelings of relief, which is often unexpected and a source of anguish for the caregiver (Nathanson & Rogers, 2020). Thus, the interaction of ambiguity and ongoing loss inherent in the dementia process may lead to worse mental health outcomes in caregivers of people with AD compared to other caregivers, resulting in an increased risk of depression, low self-esteem, social isolation, anxiety, and increased feelings of guilt and anger (Nathanson &Rogers, 2020; Dehpour & Koffman, 2022). Therefore, it is crucial to recognize and address the unique needs of family caregivers, through the dementia process, in order to provide them with adequate support and resources to navigate through their grief and maintain their health (Jain et al., 2019).

This study analyzed informal caregivers' grief and bereavement experiences, focusing on the period following the loss of their family member during the COVID-19 context. The specific aims of this study were:1-To understand the perspective of family caregivers regarding caregiving during the pandemic; 2-To understand the end-of-life phase from the caregiver’s perspective and 3-To know how family caregivers experienced the grieving process of losing their loved one with AD.

Overall, this qualitative study, may provide a deeper understanding of the unique experiences and challenges faced by family members grieving the loss of a loved one with AD. This study offers insights into the specific impact of the pandemic on the grieving process that may inform the development of targeted support strategies to meet the evolving needs of caregivers during those unprecedented times till the present. Thus, this study may contribute to understand the grieving process for family members caring for someone with AD in the context of the pandemic by exploring its unique challenges, examining coping strategies, studying its impact on mental health, identifying support needs, and informing clinical practice. To our knowledge, prior studies have yet to investigate this topic in the Portuguese context.

## Method

### Ethics

The project was approved by the Ethics Committee for Research in Life and Health Sciences from the University of Minho (Ref. CEICSH 46/2020).

A semi-structured interview consisting of open-ended questions, developed for this study, took place in the participants' homes. Before the interviews, family caregivers were informed about the study’s aims and the voluntary nature of participation by signing an informed consent form, ensuring the participant`s confidentiality. Permission to audio record was granted from all participants.

Caregiver´s interviews were conducted by a psychologist researcher. Interviews were audio-recorded, transcribed verbatim, and anonymized to guarantee the confidentiality of the data.

### Sample

Portuguese informal caregivers were recruited from the *IADem Plan* - The Dementia and Fragility Research and Action Plan in a central county in Northern Portugal. This Plan includes a research and support office for family caregivers and individuals with dementia and fragility, along with home visits.

A total of 22 IC(s) who lost their elderly family members during the first and second waves of COVID-19 in Portugal were identified and included in the initial sample. All the participants met the inclusion criteria: (1) being a grieving informal caregiver of a family member with AD in a moderate or severe stage of the disease; (2) being a user of the Portuguese National Health Care System; (3) experiencing the death of the loved one for more than six months; (4) living with a person with AD; (5) being over 18 years old. The exclusion criteria were receiving psychological or psychiatric support; having cognitive deficits (assessed by the Mini-Mental State Questionnaire, MMSE); and presenting prolonged grief disorder (assessed by Prolonged Grief Disorder-13, PG-13). As a result, 14 individuals were excluded from the study: six presented prolonged grief disorder, as assessed by the PG13 scale, and were promptly referred for psychological support. Additionally, eight caregivers showed evidence of cognitive deficits based on the MMSE. The final sample included eight caregivers.

### Measures

#### Sociodemographic Questionnaire

This instrument was designed for the quantitative study, and it assesses several sociodemographic (e.g., sex, age, marital and professional status, years of education) variables.

#### Mini Mental State Examination

Mini mental state examination was developed by Folstein et al. (1975), and adapted to the Portuguese population by Guerreiro et al. (1994). MMSE assesses cognitive function, and the maximum score is 30 points, with higher values indicating better cognitive performance. The cut-off point are the follwing: illiterates ≤15; 1–11 years of education ≤22; more than 11 years of education ≤27). This instrument was used as a screening tool for cognitive impairment and dementia (Santana et al., 2016).

#### Prolonged Grief Disorder-13

Prolonged grief disorder-13 was developed by Prigerson and Maciejewski (2006) based on consensus criteria for diagnosing Prolonged Grief Disorder. The instrument was developed as a screening tool to identify individuals experiencing prolonged and complicated grief reactions. It consists of 13 descriptive items that assess symptoms that must persist for a minimum period of 6 months and are necessarily associated with significant functional impairment. The first part of the questionnaire comprises two items that assess the frequency of separation anxiety, answered in a Likert scale, ranging from 1 (Almost never) to 5 (Several times a day). The third item refers to the duration of the symptom, with a dichotomous: affirmative answer if the symptom has lasted equal to or longer than six months, and a negative answer if the manifestation persited less than six months. The second part consists of nine descriptive items assessing cognitive, emotional, and behavioral symptoms answered in a Likert scale, ranging from 1 to 5, indicating the intensity of each symptom. The final question pertains to functional impairment in social, occupational, or other domains of functioning, answered in a dichotomous form (yes or no). A total score is calculated summing all the answers. Higher scores indicate a higher level of prolonged grief symptoms. PG-13 showed high internal consistency (Cronbach’s alpha was 0.93) in the original and Portuguese versions.

### Interview Guide

A semi-structured interview with open-ended questions (Table 1) was used to collect caregivers’ perceptions of grief regarding their family member with AD. The script did not change throughout the interviews. The first section concerned the experience of caring in the context of a pandemic; the second section addressed the risk of contracting COVID as a caregiver and whether it influenced the caregiving; the third section concerned the moment and circumstances of death, and the last one focused on the process of grieving.

**Table 1.** Open-Ended Interview Frame for Bereaved Family Caregivers.

1- What was it like (and continues to be) to experience grief in the context of a pandemic?
2- How was it to reorganize your life after his/her passing?
3- Who did you talk to or seek/receive support from during that time?
4- When did you realize that death was inevitable?
5- How did the risk of contracting COVID-19, as a caregiver, influence the care you provided?
6- What were the positive and negative aspects of your experience as a caregiver during the COVID-19?
7- What changed in your caregiving routine due to COVID-19 in your family?
8- How did you feel when providing care?
9- Now looking back, what would you have done differently, if anything?

### Data Analysis

The study followed the consolidated criteria for reporting qualitative research based on the COREQ checklist (Tong et al., 2007). A 6 months post-death for bereaved participants was chosen since it is approximately when normal grief transitions are integrated into healthy grief (Miller et al., 2020).

The transcriptions were analyzed in the same order they were conducted, using the thematic analysis technique through NVivo software (QSR International Pty Ltd. NVivo, 2023). To address the objectives of the qualitative study, the thematic content analysis technique was used (Bardin, 2016). A postpositivist paradigm was adopted (Ponterotto, 2005), and thematic content analysis was selected since it enables a comprehensive and profound understanding of participants' perceptions regarding the grieving process. This approach is characterized by its rigor and systematic nature, allowing for a structured and in-depth data exploration. The coding process of the transcripts involved an inductive approach, where two independent coders analyzed the data and identified subthemes. The coders discussed their coding and grouped the subthemes into overarching themes. Higher-order domains were established based on the study’s objectives, ensuring alignment with the research aims and objectives. The coding and theme development process followed a rigorous and systematic approach to ensure the accuracy and reliability of the findings.

## Results

The sample included eight female informal caregivers of an elderly family member with AD who passed away at least six months prior to the interview. The characterization of the sample is presented in Table 2.

**Table 2.** Demographic Characteristics of the Individuals With AD (*n* = 8) and Their Informal Caregivers (*n* = 8).

Continuous variables	Family Caregivers				Person living with AD			
	Min	Max	Medium	SD	Min	Max	Medium	SD
Age	45	77	57.12	10.66	80	95	87.62	5.42
Education	2	5	3.88	.835	0	2	.5	.93
Duration of care (years)	6	15	9	3.16				

Categorical Variables	Frequency	%	Frequency	%
Sex				
Male			3	37.5
Female	8	100	5	62.5
Marital status				
Married	6	75	3	37.5
Unmarried	2	25		
Widowed			5	
Degree of kinship				
Parents	5	62.5		
In-laws	3	37.5		
1st time caregiver				
Yes	8	100		
Laboral situation				
Active	4	50		
Retired	4	50		

**Figure 1.**
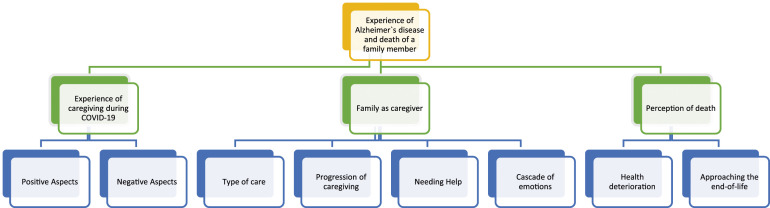
Results from the analysis of caregivers’ interviews about “Experience of AD and the death of a family member.”

**Figure 2.**
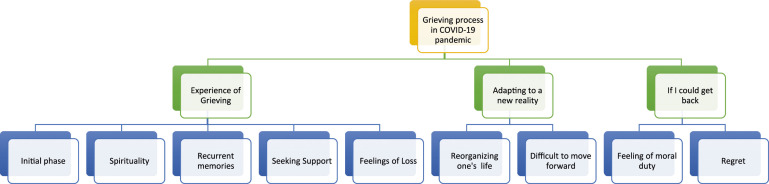
Results from the analysis of caregivers’ interviews about “Grieving process during the COVID-19 pandemic.”

Two higher-order domains were found: – “Experience of AD and the death of a family member” (Figure 1 and Table 3) and the “Grieving process during the COVID-19 pandemic” (Figure 2 and Table 4).

**Table 3.** Domain 1 - Experience of AD and the Death of a Family Member.

Experience of Caregiving during the COVID19	Positive Aspects	“Look, there were positives… there were some, right? As a caregiver, I took care… and I always feel that I took great care of my mother… cleaning ...everything”.
		“Well, I always took care of her as best as I could”.
		“Learning how to better care for someone who was bedridden because I did not have any training, none at all”.
		“I had to learn how to give a bath in a different way, bathing in bed”. “Also being patient and flexible and try to adapt to your loved one’s changing needs for not contracting COVID-19”.
		“I never transmitted anything to my father, neither COVID-19 nor anything else, because I was always careful”.
	Negative aspects	“Because she was used to them coming in and they would always give her a little kiss, show her affection… and at that time, we had to prepare her a little for not receiving”.
		“Having to fully equip oneself to take care of someone, like wearing masks and gloves, and being cautious…we always wore masks and were well protected, and no one would come in here… if someone came to see him, they would also wear masks…”.
		“With a lot of fear… at that time, I was very afraid of contracting COVID-19 and then passing it on to my mother. I was really scared. There were times when I stayed at home and did not go out. Only when it was necessary… because I was really scared… but a lot has changed… even so, during the time of COVID-19, I stopped doing many things because I was afraid… so, I was always anxious about catching it, and I was scared.”
		“It is only when he needs care provided by others… that was a bad sign, right?”.
Family as caregiver	Type of care	“She was practically, completely dependent… in terms of self-care”.
		“Having to do everything, wash her, dress her, take care of all hygiene”.
		“I did not work or anything, I was always here at home and even outside, but always around, and we were always worried… we would always come to give her a snack because she could not eat by herself”.
	Progression of caregiving	“She got worse after receiving the COVID-19 vaccine… her legs weakened a lot, she could not walk anymore, even though she could still walk a little before”.
		“She would go to the hospital almost every week… sometimes even every other day because she had difficulty breathing”.
	Needing help	“I helped with changing her”.
		“That was something I could not do, and if she could not do it, two girls and my daughter would come”.
		“When I knew that he was feeling a bit down, struggling to breathe, I would already call him, and he would come”.
	Cascade of emotions	“Well, I managed to go all the way without asking for help from anyone… but I will tell you something, I was already reaching my limit”.
		“I felt anguished… that is what it was… day by day, you could see her getting worse”.
		“And with the person she wanted, which was me”.
		“That is what I could give her… it was love and affection… when I arrived, when I arrived… she would say, come here, my daughter… being satisfied to say that I am the one taking care of my mother”.
Perception of death	Heath deterioration	“She was in a vegetative state, so she did not speak, did not communicate, did not recognize anyone”.
		“She passed away in the hospital… she stayed in the hospital during the second admission because she had acquired… she had pneumonia”.
		“She could not speak anymore, she was getting worse and worse… then she started to deteriorate, and when I went to see her during that half-hour that was allowed, she could not longer speak … she did not react to my voice”.
	Approaching the end-of-life	“It was a bit difficult because at that time, of course, I was aware that she, my mother, was very ill, and that death was inevitable at any moment”.
		“You can do a lot in terms of comfort… so that she was very comfortable… all the comfort that I could provide”.
		“But the last time, when they removed and took everything off, (clinical devices) we could see that he was not going to come back”.

**Table 4.** Domain 2 - Grieving Process During the COVID-19 Pandemic.

Experience of Grieving	Initial phase	“In the beginning, it was difficult, and I felt it deeply”.
		“It was during that month or two that I felt quite shaken”.
		“When everything is still very fresh, it is a different kind of pain”.
		“That is how it was when I received the phone call saying that she had passed away, honestly, at that moment, I could not even process that she had died”.
		“I could not believe it, you know?”.
	Spirituality	“She was very religious, a devout catholic, always holding her rosary in her hand for everything, and it was not just any funeral… it was a very sad funeral, and we were sad because she was very devoted… because my mother did not have her body presence during the funeral liturgy”.
		“Even in mourning, we went through the funeral, everything according to the rules, not for us, the family, but more for those who were there, as we, the family, were always together, and we did not have that problem of restrictions… it was more the people coming from outside”.
		“Yes, at the church, there were restrictions, that is how it was… but at the funeral home, no… we had the freedom, as a family, to say our goodbyes to her as it should be”.
		“It went well… it was resolved quickly… thank God everything went well, and there were no restrictions or anything”.
	Recurrent memories	“He was good company, you know… It’s hard…he was a saint, even regarding his illness”.
		“Sometimes I would jump out of bed and… I would go straight to bed again because it was that habit of always being there, whether she called or not… always having that thought”.
		“Life goes on, but he is always there, present everywhere… sometimes when I am sitting there… I feel like my father is all around me… I have a friend”.
		“…I look at the things he loved, anything he did… even today, I cannot untie a simple wire that he tied… I try to do what he loved most in life and take care of his things, which are now in our hands… now he is already here to see it all, but his memory will always be there, and I try to do what he liked, what he did the most”.
	Seeking support	“My sister came here and never left”.
		“I also spoke with many people… I had many people who supported me”.
		“I also had a cousin who I took care of and provided support to”.
		“With family, with friends”.
		“Friends… they would always call me, always come to me, always listen to me”.
		“Seek professional help”.
		“Later on, I had the support of the team from palliative care, they would always call me… if I needed a psychologist, if I needed someone to talk to”.
		“The nurses from palliative care called, wrote, and called again later”.
		“The support I truly received was from my children”.
		“You know, I never had support from anyone… we find strategies to deal…”.
	Feelings of loss	“I felt bad… I felt very sad… I went through a depression (voice interrupted) I was deeply shaken”.
		“It is always very sad to lose someone we love”.
		“That pain is a pain that we have more time to think about…”.
		“There are moments, very, very difficult moments. (Cried)”.
		“There are hours, many, many difficult hours”.
		“Just to see how she is doing and everything, and there is an empty space, you know?”.
		“The feeling of missing someone continues, it still hurts a lot, and it hurts a lot… his absence…”.
		“…Nothing, I felt so empty… I missed him so much…”.
		“Because my house is a big house, and I was used to have my house full… but then it became empty”.
		“That emptiness is hard to understand… It is difficult to explain… very difficult”.
		“A great longing”.
Adapting to a new reality	Reorganizing one’s life	“Right after my mother passed away, I was very involved in that… I kept myself busy with other work, trying not to think too much”.
		“I stayed home more often and then started helping my parents more and going to their place more frequently”.
		“I tried to go out more to distract myself from thinking about her… I avoided being at home”.
		“Now I take care of my grandchildren and taking care of two other people as well”.
	Difficult to move forward	“That is why I have not finished mourning yet because we cannot”.
		“That is why I have not taken off my mourning clothes yet because we cannot”.
		“It gives me the impression that she will come back and… It is something…”.
		“I think, I do not know… they should have done more… I still have this idea, and no one can take it away from me… truthfully, it is still not well organized. I have not… it feels like I am still stumbling around, going from one place to another”.
If I could get back	Feeling of moral duty	“We did everything, we asked the physician to come over when she had a rough night… and the physician advised us to go… she did not come again, but we did everything we could”.
		“… I did everything I could, everything within my reach… I could not give her a life that was not in my hands, but I did everything that was needed”.
		“I do not think I could have done more… looking back, I do not think so… we could not do more for my father”.
		“I did what I had to do from the beginning to the end”.
	Regret	“Yes, having more patience with her… not losing my temper so easily”.
		“For example, I bought an adjustable bed, I bought a reclining chair, and those things… I should have bought earlier”.
		“I should have brought her home for the last 10 days… because I knew she wanted to spend her final days here”.

### Domain 1: Experience of AD and the Death of a Family Member

From the analysis of family caregiver’s interviews, three themes emerged that contributed to the experience of AD and the death of a family member: (a) Experience of Caregiving during the COVID-19, (b) Family as a Caregiver and (c) Perception of Death.

#### Theme a) Experience of Caregiving During the COVID-19

Taking care of a family member living with AD may be challenging even under normal circumstances. However, the pandemic COVID-19 has added an extra layer of stress and difficulties to caregiving. This theme is composed of two subthemes: *Negative Aspects and Positive Aspects*.

Negative aspects were associated with ensuring the safety of both the caregiver and the person with AD. Caregivers need to take extra precautions to protect their loved ones from exposure to the virus. This involved restriction of contact: “*Because she was used to them coming in and they would always give her a little kiss*, *showing their affection*… *and at that time, we had to prepare her a little for not receiving”;* also, protective behaviors against COVID-19: *“Having to fully equip oneself to take care of someone, like wearing masks and gloves, and being cautious*… *we always wore masks and were well protected, and no one would come in here… if someone came to see him, they would also wear masks… people did not walk around without masks”*. Fear was also another negative aspect: *“At that time, I was very afraid of contracting COVID-19 and then passing it on to my mother… I was really scared… there were times when I stayed at home and did not go out…only when it was necessary, because I was really scared…* *but a lot has changed… even so, during the time of COVID-19, I stopped doing many things because I was afraid… I was always anxious about catching it, and I was scared”* and, finally, the fact of being dependent of the caregiver “*It is only when he needs care provided by others… that was a bad sign, right?”*.

Positive aspects were described like being a caregiver for a family member with AD as a challenging and emotional positive experience. AD can cause unpredictable changes in behavior and mood, and caregiver must have a sense of duty *“Look, there were positives… and… there were some, right? As a caregiver, I took care… and I always feel that I took great care of my mother… cleaning everything”*. Being a good caregiver “*Well, I always took care of her as best as I could”*; *“Learning how to better care for someone who was bedridden because I didn’t have any training, none at all”*. New learnings *“I had to learn how to give a bath in a different way, bathing in bed”; “Also, being patient and flexible and try to adapt to your loved one’s changing needs for them to be protected and not contract COVID-19”*; *“I never transmitted anything to my father, neither COVID-19 nor anything else, because I was always careful”*.

#### Theme (b) Family as Caregiver

This theme is composed of four subthemes:* Type of Care; Progression of Caregiving; Needing Help; *and *Cascade of Emotions*.

The type of care needed for a family member living with AD depends on the stage and severity of the disease. Some common types of care were described: personal care - involving helping with activities of daily living, such as bathing, dressing, grooming, and toileting and medication management *“She was practically, completely dependent… in terms of self-care”*; *“Having to do everything, wash her, dress her, take care of all hygiene”*. Another type of care was emotional support by offering reassurance, comfort, and companionship *“I did not work or anything, I have been always here at home and even outside, but always around, and we were always worried… we would always come to give her a snack because she could not eat by herself”*.

Caring for a family member with AD may be challenging and emotionally demanding. As the disease progresses, the care needs of the individual may increase, requiring more time, resources, and support. In the early stages of Alzheimer’s, the care may involve monitoring the individual’s safety, assisting with activities of daily living, and providing companionship: *“She got worse after receiving the COVID-19 vaccine… her legs weakened a lot, she could not walk anymore, even though she could still walk a little before”*. As the disease progresses, caregiving may involve managing behavior changes, medication management, and addressing medical needs such as infections, injuries, and chronic conditions: *“She would go to the hospital almost every week… sometimes even every other day because she had difficulty breathing”*.

When caring for a family member, there is often a primary caregiver, who takes on most of the caregiving responsibilities, including personal care, managing medications, and providing emotional support. When this caregiver needs help, he/she seeks for someone to provide additional support and assistance: *“I helped with changing her”; “That was something I could not do, and if she could not do it, two girls and my daughter would come”; “When I knew that he was feeling a bit down, struggling to breathe, I would already call him, and he would come”*.

Caregiving may evoke a cascade of emotions including overwhelming feelings- the demands of caregiving can be overwhelming, especially when balancing other responsibilities *“Well, I managed to go all the way without asking for help from anyone… but I will tell you something, I was already reaching my limit”*. Sadness and anguish also emerged: “*It is natural to feel sadness as the disease progresses and the person loses their abilities and memories”; “I felt anguished… that is what it was… day by day, you could see her getting worse”*. Loss of identity was also mentioned. Caregivers may feel like their identity had become solely defined by their caregiving role, leading to a loss of individuality and autonomy: *“And with the person she wanted, which was me”*. Also, caregiving for a family member with AD may evoke a range of positive emotions such as a sense of purpose and fulfillment, increased empathy, and a stronger sense of connection with the person being cared for: “That is *what I could give her… it was love and affection. When I arrived, she would say, come here, my daughter… being satisfied to say that I am the one taking care of my mother”*.

#### Theme c) Perceptions of Death

This theme had two subthemes: *Health Deterioration and Approaching the End-of-Life.*

Health deterioration of a family member with AD may be emotionally and physically challenging for informal caregivers. As the disease progresses, the person with AD may become increasingly dependent on their caregiver for activities of daily living, such as eating, dressing, and toileting *“She was in a vegetative state, so she did not speak, did not communicate, did not recognize anyone”*. Additionally, caregivers may need to adjust the care plan as the AD progresses. Progresses, involving seeking out new resources, such as in-home care or assisted living facilities, as the level of care required becomes more intensive: *“She passed away in the hospital… she stayed in the hospital during the second admission because she had acquired… she had pneumonia”*; *“She could not speak anymore, she was getting worse and worse… then she started to deteriorate, and when I went to see her during that half-hour that was allowed, she could not longer speak … she did not react to my voice”*.

Approaching the end-of-life, the caregiver’s role shifted in order to provide comfort and palliative care, including pain management, emotional support, and spiritual care *“It was a bit difficult, difficult, because at that time, of course, I was aware that she, my mother, was very ill, and that death was inevitable at any moment”; “You can do a lot in terms of comfort… so that she was very comfortable… all the comfort that I could provide”; “But the last time, when they removed and took everything off (clinical devices), we could see that he wasn’t going to come back”*.

### Domain 2 - Grieving Process During the COVID-19 Pandemic

From the analysis of informal caregiver’s interviews, three themes emerged that contributed to the grieving process in a COVID-19 pandemic: (a) *Experience of Grieving, *(b) *Adapting to a New Reality* and (c) *If I Could Get Back.*

#### Theme a) Experience of Grieving

This theme had five sub themes: Initial phase, Spirituality, Recurrent Memories, Seeking Support, and Feelings of Loss. The initial phase may be characterized by shock, denial, disbelief, and a sense of numbness. The caregiver may feel overwhelmed by the diagnosis and unsure of how to move forward. *“In the beginning, it was difficult, and I felt it deeply”; “It was during that month or two that I felt quite shaken”; “When everything is still very fresh, it is a different kind of pain”; “*That is *how it was when I received the phone call saying that she had passed away, honestly, at that moment, I could not even process that she had died”; “I could not believe it, you know?”*.

Grief may become a deeply spiritual experience, especially when associated with the coping with the loss and the behavioral changes of the family member with AD. Spirituality may provide comfort, meaning, and a sense of connection during a difficult time. In COVID-19, some catholic rituals were different “*She was very religious, a devout Catholic, always holding her rosary in her hand for everything, and it was not just any funeral… it was a very sad funeral, and we were sad because she was very devoted… because my mother did not have her* *body presence during the funeral liturgy”; “Even in mourning, we went through the funeral, everything according to the rules, not for us (the family) but more for those who were there, as we, the family, were always together, and we did not have that problem of restrictions…it was more for the people coming from outside”; “Yes, at the church, there were restrictions,* that is *how it was…but at the funeral home, no… we had the freedom, as a family, to say our goodbyes to her as it should be”*; *“It went well…it was resolved quickly….thank God everything went well, and there were no restrictions or anything”*.

Recurrent memories of a family member with AD when grieving may be both positive and negative and can evoke a range of emotions. *“He was good company, you know… it is hard… he was a saint, even regarding his illness”; “Sometimes I would jump out of bed and… I would go straight to bed again because it was that habit of always being there, whether she called or not… always having that thought”*; *“Life goes on, but he is always there, present everywhere… sometimes, when I’m sitting there… I feel like my father is all around me. I have a friend”*; *“… I look at the things he loved, anything he did… even today, I cannot untie a simple wire that he tied… I try to do what he loved most in life and take care of his things, which are now in our hands… now he is already here to see it all, but his memory will always be there, and I try to do what he liked, what he did in the most”*.

Grieving the impact of AD on a family member may be a complex and challenging process. Seeking support during this time is very important. Reach out to family and friends were very common strategies: *“My sister came here and never left”; “I also spoke with many people… I had many people who supported me”*; *“I also had a cousin who I took care of and provided support to”*; *“With family, with friends”*; *“Friends would always call me, always come to me, always listen to me”*. Also, seek professional help: *“Later on, I had the support of the team from palliative care, they would always call me… if I needed a psychologist, if I needed someone to talk to”; “The nurses from palliative care called, wrote, and called again later”*. Some informal caregivers reported: *“The support I truly received was from my children.”*; *“You know, I never had support from anyone… we find strategies to deal”*.

Feelings of loss arise from grieving the loss of a loved one to AD, also emerged. Some common feelings that IC’s experienced were sadness: *“I felt bad… I felt very sad… I went through a depression (voice interrupted) I was deeply shaken”; “It is always very sad to lose someone we love”*. Pain and suffering were also reported: *“That pain is a pain that we have more time to think about…”*; *“There are moments, very, very difficult moments (crying).”*; “*There are hours, many, many difficult hours”*. Feeling of emptiness during the process: *“Just to see how she is doing and everything, and there is an empty space, you know?”; “The feeling of missing someone continues, it still hurts a lot, and it hurts a lot… his absence…”; “… Nothing, I felt so empty… I missed him so much…”*; *“Because my house is a big house, and I was used to have my house full, but then it became empty”*; *“That emptiness is hard to understand… it is difficult to explain, very difficult”*. Finally, the experience of longing was reported:* “A great longing”*.

#### Theme (b) Adapting to a New Reality

This theme includes two subthemes: *Reorganizing Life *and* Difficulties to Move Forward.*

Reorganizing one’s life after the loss of a family member in AD can be a significant adjustment. Family caregivers described adjusted routines and responsibilities *“Right after my mother passed away, I was very involved in that… I kept myself busy with other work, trying not to think too much”*; *“I stayed home more often and then started helping my parents more and going to their place more frequently”*; *“I tried to go out more to distract myself from thinking about her… I avoided being at home”*; *“Now I take care of my grandchildren and taking care of two other people as well”*.

For some caregivers, the process of grieving became lengthy and intense, significantly impairing an individual’s ability to function and making it difficult to move forward. Family caregivers described some situations: “That is *why I have not finished mourning yet because we cannot”; “*That is *why I have not taken off my mourning clothes yet because we cannot”; “It gives me the impression that she will come back and… it’s something…”; “I think, I do not know… they should have done more… I still have this idea, and no one can take it away from me… truthfully, it is still not well organized… I have not… it feels like I am still stumbling around, going from one place to another”*.

#### Theme c) If I could Go Back

This theme had two subthemes:* Feelings of Moral Duty* and *Regret.*

A fulfilled feeling of moral duty and the grieving process are often intertwined in caregivers of family members with AD. Caregiving often involves a strong sense of responsibility and devotion to the loved one’s well-being as illustrated by one of the participants regarding the overwhelming emotions of duty and grief *“We did everything, we asked the physician to come over when she had a rough night… and the physician advised us to go… she did not come again, but we did everything we could”; “… I did everything I could, everything within my reach… I could not give her a life that was not in my hands, but I did everything that was needed”*; *“I do not think I could have done more… looking back, I do not think so… we could not have done more for my father”; “I did what I had to do from the beginning to the end”*.

Regret is a common and complex emotion that caregivers may experience when grieving the loss of a family member with AD emerging from the pressure associated with unrealistic standards: “*Yes, having more patience with her…not losing my temper so easily”; “*For example*, I bought an adjustable bed, I bought a reclining chair, and those things… I should have bought earlier”*; *“I should have brought her home for the last 10 days, because I knew she wanted to spend her final days here”*.

## Discussion

This qualitative study aimed to explore the experiences of caregivers of elderly family members with AD during the COVID-19 pandemic and their grieving process. The caregivers in this study were predominantly women, primarily daughters, with an average age of 57 years old, half of them still working, and with an average duration of caregiving of approximately nine years. These demographic characteristics align with previous research findings reported in both national and international studies (Costa et al., 2019; Crawley et al., 2022). Two higher-order domains were identified from the interviews. The first domain, “Experience of AD and the death of a family member,” was further explored through three themes: (1) “Experience of caregiving during the COVID-19,” highlighting the challenges and adaptations caregivers faced in providing care amidst the pandemic; (2) “Family as caregivers,” illustrating the dynamics of family caregiving and the impact on the caregivers' emotional well-being; and (3) “Perception of death,” exploring the caregivers' reflections on the death of their loved ones and its significance in their lives. The second domain, “Grieving process during the COVID-19 pandemic,” was also characterized by three themes: (1) “Experience of Grieving,” revealing the emotional journey of grief and the range of emotions experienced by the caregivers; (2) “Adapting to a new reality,” highlighting how the pandemic influenced the grieving process and the caregivers' efforts to cope with loss amidst the pandemic challenges; and (3) “If I could get back,” reflecting on the caregivers' feelings of regret and longing for more time with their deceased family members.

Regarding the experience of caregiving during the COVID-19, the study findings suggested that individuals engage in family caregiving for several reasons. Caregivers report as positive aspects of their caregiving, a desire to protect and improve the well-being of the person in need, new competencies, a sense of duty to repay the care received during childhood, and the extension of caring roles within affective relationships. These motivations highlight the importance of personal connections, responsibilities, positive gains, and a sense of duty in assuming the caregiver role, as described in some studies (Yu et al., 2018). These results emphasize family caregiving as a positive experience, leading to personal accomplishment and satisfaction, feelings of mutual support in a relationship, improved family cohesion and functionality, and a sense of personal growth and purpose in life (Mason et al., 2020). Also, negative challenges were reported, such as limited access to support services, restrictions on visitation, and increased isolation, fear, and protective behaviors against COVID-19 impact negatively. The results are consistent with previous studies focused on COVID-19. However, the present study was conducted with older adults and caregivers from a community sample (Navaei Kolvanagh et al., 2023; Petzold et al., 2020).

Family caregiving described how informal caregivers provide a significant amount of care, dedicating more hours per week and assisting with a more significant number of activities of daily living (ADL) or instrumental activities of daily living (IADL) (Karg et al., 2018), especially in severe AD. As the disease progresses, the person living with AD may require around-the-clock care, including assistance with all activities of daily living, feeding, and toileting, as described by participants. In the present study, both primary and secondary caregivers communicated effectively and worked together as a team to provide the best possible care. In some cases, caregivers sought outside help, such as hiring a professional caregiver or health professional belonging to the IADem plan, when some decision had to be taken, did not feel well or needed help to support the end of life and grieving process.

AD, being a terminal illness, leads caregivers to engage in a grieving process over time as both the person living with AD and the caregiver experience ongoing losses over an extended period. The perception of death, described by the participants, as a release from the worsening health state adds to the complexity of the grieving experience for caregivers (Robertson & Kagee, 2023). The findings suggest that caregivers often must adjust the care plan as the disease progresses. According to the results, caregivers strive to provide comfort and palliative care, seeking support from health and social resources to ensure the best care for their elderly loved ones. This adaptive approach is consistent with the existing literature on advanced care planning, emphasizing the importance of proactive decision-making in the face of dementia and other terminal illnesses (Mogan et al., 2022). Advanced care planning has been shown to have several positive outcomes for caregivers. One notable benefit is choosing the prefered place to die. By engaging in discussions about end-of-life care preferences, caregivers can ensure that their loved one’s wishes are respected and that they receive care in the environment they desire most. This is particularly important in AD, where the person’s ability to communicate their preferences may decrease as the disease progresses.

Moreover, when caregivers actively participate in advanced care planning and are aware of their loved one’s preferences, some studies reported higher levels of satisfaction with the care provided (Dassel et al., 2023). In this study, caregivers’ satisfaction arises from the perception of fulfilling their loved one’s wishes and providing care that aligns with their values and beliefs. Such alignment may contribute to a sense of fulfillment and reduce the guilt or uncertainty that caregivers may experience when making crucial decisions on behalf of their loved ones. Additionally, some studies (Dixon et al., 2018; Dwyer et al., 2015) evidenced that when caregivers were well-informed about the person’s preferences regarding caregiving, they could anticipate and address potential crises more effectively.

Regarding the experience of grieving, in this study, participants expressed initial shock, disbelief, and emotional numbness as a protective response to the loss, in the initial phase, which is in accordance with John Bowlby’s attachment theory (1979) and Parkes' four-phase model of grieving (1998). These theoretical frameworks provide valuable insights into the experience of grief and loss, highlighting the emotional and psychological responses individuals may go through the initial stages of bereavement. This study’s findings align with those of Mazzaschi et al. (2023) and Skantharajah et al. (2021), highlighting the impact of the COVID-19 pandemic on the grieving process and mourning rituals. However, most participants relied on religiosity and spirituality before and during mourning, reporting a good relationship with the person they cared for, having access to family, friends, and community support, and accepting the impending death.

Caregivers also described grief as a natural and complex emotional response characterized by a range of feelings that may include shock, pain, and suffering, a feeling of emptiness, sadness, anger, and longing for the lost person relating to spiritual and religious beliefs. Using religion and spirituality as coping strategies appears to be beneficial since religious practices, beliefs, and attendance have been associated with improved mental health and decreased psychological distress (Britt et al., 2023; Giannouli & Giannoulis, 2020). Furthermore, in the present study, caregivers placed emphasis on quality time and spiritual engagement, reporting their faith offered them valued support, fostering reciprocal relationships. The implementation of collaborative spiritual coping may empower caregivers by facilitating post-traumatic spiritual growth following bereavement (McGee et al., 2022).

As AD progresses and losses accumulate, caregivers may experience prolonged and compounded grief reactions. The emotional toll of caring for individuals with AD or dementia has been widely documented in other studies, including those by Rodríguez-Mora et al. (2023) and Williamson et al. (2020). The pandemic added more challenges, as caregivers face increased isolation and limitations on their usual support systems. This high emotional burden may negatively impact caregivers' mental health and well-being. It is essential to provide a supportive environment, like family and friends or seek professional help, where caregivers may freely express their emotions (Jain et al., 2019). In this context, the present study provides an understanding about the importance of maintaining connections with their deceased loved ones, engaging in shared memories, and taking care of things the deceased loved, helped in preserving and cherishing the bond, such as the Continuing Bonds Theory suggests (1996). Also, the recurrent memories reported by caregivers are in accordance with the meaning reconstruction theoretical model (Gillies & Neimeyer, 2006) that emphasizes the importance of finding meaning in the caregiving role, the closeness and warm relationship with their loved one, and the lessons learned through the experience of caring that provided a sense of purpose and integrated the loss into their life narrative.

## Health Care Implications

According to the results, encouraging caregivers to engage in activities that allow them to continue their relationship with the deceased in meaningful ways may also be beneficial for their grieving process. These activities may trigger new memories and provide different perspectives, facilitating the integration of the loss into their lives. So, allowing caregivers to express their emotions and engage in meaningful activities related to their deceased loved ones is crucial. Empowering caregivers to focus on what they can control may help their grieving process and the adaptation to the changes in their lives.

The grief experienced by caregivers is a profoundly personal and individual process, requiring time and compassion to navigate effectively. There is no one-size-fits-all approach to grieving, and caregivers must be gentle with themselves as they cope with the loss of their loved ones. Each person’s grief journey is unique, and acknowledging this diversity may help caregivers feel more supported and validated in their experiences. Professionals involved in bereavement services and counseling play a pivotal role in providing comprehensive care to those experiencing loss. Nurses, psychologists, social workers, and other healthcare providers must address the physical, psychosocial, and spiritual needs of bereaved individuals and assist caregivers in coping with their grief through open and sensitive end-of-life discussions like end-of-care planning and ongoing interventions.

Moreover, bereavement interventions need to consider the factors that mediate grief, such as the specific experiences related to chronic health conditions, cultural practices, and the care setting. Tailoring support to these unique circumstances and providing compassionate and personalized support may enhance the efficacy of bereavement interventions and help caregivers find meaning and healing in their grieving process. Also, our findings suggest that the identity as a caregiver may be a positive continuum of life and help caregivers to reorganize their lives, since caregiving provides a sense of duty and responsibility, which offers meaning to the experience. Additionally, caregiving may foster a sense of community and connection with others, which is especially important during a time of crisis like the pandemic context.

The present study also sheds light on the complex and emotional nature of grieving experienced by caregivers after the loss of their loved ones with AD. The implications for practice emphasize the importance of addressing caregiver's identity, fostering social support and acceptance, allowing the expression of emotions and promoting spiritual and psychological well-being. Intervention should encourage caregivers to engage in meaningful activities that trigger new memories and provide different perspectives, offering grief support, and empower caregivers to focus on what they can control through realistic goals, plans for the future, and palliative care. Therefore, healthcare professionals are instrumental in facilitating healthy grieving processes and enhance caregivers' coping strategies during bereavement.

## Limitations

This study is not without limitations, which warrant careful consideration. One significant limitation is the reliance on the subjective perceptions of a relatively small number of participants. Furthermore, the study used a convenient sample, and a cross-sectional design rather than studying the caregivers' grief responses over time.

As a result, the findings should be interpreted cautiously. Furthermore, the thematic analysis of the interviews showed a degree of coherence in the emerging themes. Despite these limitations, this study provides valuable insights into the grieving process of IC(s) caring for a family member with AD, highlighting the emotional challenges and coping strategies that caregivers encounter, shedding light on the complexities of the caregiving journey and bereavement experiences. Future quantitative studies should test these results in larger samples with more diverse participant cohorts.

## Conclusion

One of the significant findings reported by caregivers was their identification with the caregiving role: after the loss, their identity remained unchangeable. This insight sheds light on the enduring impact of caregiving on their identity and sense of self. The findings suggest that caregivers may continue to experience a sense of purpose and connection to their caregiving role even after their loved ones have passed away. This ongoing identification as a caregiver may influence their coping strategies and emotional responses to grief, as was evidenced in some studies (Beatie et al., 2021) and may also shape caregivers' perceptions of the caregiving journey and the meaning they ascribed to their experiences.

The results also showed that family caregivers, in order to mitigate their loss, developed new routines and meaningful activities, stimulated social support, tolerated ambiguity, encouraged hope, had recurrent memories, and discovered a sense of meaning in spirituality that provided significance by continuing the relationship in a meaningful way while still mourning. Therefore, the development and implementation of interventions regarding the grief process must consider the substantial investment of the caregiver’s acceptance and commitment towards the person they lost to yield successful outcomes. Moreover, the grief experienced by family caregivers of a person living with AD is a multifaceted phenomenon that needs to acknowledge cultural, familial, individual, and environmental factors.
